# Promoter hypermethylation of the tumor-suppressor genes *ITIH5, DKK3*, and *RASSF1A *as novel biomarkers for blood-based breast cancer screening

**DOI:** 10.1186/bcr3375

**Published:** 2013-01-15

**Authors:** Vera Kloten, Birte Becker, Kirsten Winner, Michael G Schrauder, Peter A Fasching, Tobias Anzeneder, Jürgen Veeck, Arndt Hartmann, Ruth Knüchel, Edgar Dahl

**Affiliations:** 1 Molecular Oncology Group, Institute of Pathology, Medical Faculty of the RWTH Aachen University, Pauwelsstrasse 30, D-52074 Aachen, Germany; 2 Department of Gynecology and Obstetrics, University Hospital Erlangen, Universitätsstrasse 21-23, D-91054 Erlangen, Germany; 3 Patients' Tumor Bank of Hope (PATH) Foundation, Schäftlarnstr. 62, D-81337 München, Germany; 4 Department of Pathology, University of Erlangen, Krankenhausstrasse 12, D-91054 Erlangen, Germany

## Abstract

**Introduction:**

For early detection of breast cancer, the development of robust blood-based biomarkers that accurately reflect the host tumor is mandatory. We investigated DNA methylation in circulating free DNA (cfDNA) from blood of breast cancer patients and matched controls to establish a biomarker panel potentially useful for early detection of breast cancer.

**Methods:**

We examined promoter methylation of seven putative tumor-suppressor genes (*SFRP1*, *SFRP2*, *SFRP5, ITIH5*, *WIF1*, *DKK3*, and *RASSF1A*) in cfDNA extracted from serum. Clinical performance was first determined in a test set (*n *= 261 sera). In an independent validation set (*n *= 343 sera), we validated the most promising genes for further use in early breast cancer detection. Sera from 59 benign breast disease and 58 colon cancer patients were included for additional specificity testing.

**Results:**

Based on the test set, we determined *ITIH5 *and *DKK3 *promoter methylation as candidate biomarkers with the best sensitivity and specificity. In both the test and validation set combined, *ITIH5 *and *DKK3 *methylation achieved 41% sensitivity with a specificity of 93% and 100% in healthy and benign disease controls, respectively. Combination of these genes with *RASSF1A *methylation increased the sensitivity to 67% with a specificity of 69% and 82% in healthy controls and benign disease controls, respectively.

**Conclusions:**

Tumor-specific methylation of the three-gene panel (*ITIH5*, *DKK3*, and *RASSF1A*) might be a valuable biomarker for the early detection of breast cancer.

## Introduction

Breast cancer remains the most frequently diagnosed cancer and the leading cause of cancer deaths in European women [1]. According to the World Health Organization (WHO), more than 449,000 women in Europe will be diagnosed yearly with breast cancer, comprising approximately 28% of all cancers in female patients. Localized, early-stage breast cancer has a favorable prognosis, with a 5-year survival rate of up to 98%. However, the 5-year survival rate declines to 20% when the tumor has metastasized [2]. Although clinical examination and ultrasound provide a preliminary screening method, the two most sensitive and specific detection methods to date are mammography and magnetic resonance imaging (MRI). Mammography has become the standard of care in breast cancer screening. Mammograms are useful in that they are reliable and sensitive enough to detect ductal carcinoma *in situ *(DCIS). Nevertheless, the limitations of mammography are well recognized [3,4], such as the personal discomfort, the poor accuracy in women with dense breast tissue, and a relatively high rate of false positives. Clinically occult early-stage breast cancer is often similar in appearance to a benign breast lesion, resulting in unnecessary subsequent biopsies [3]. In addition, more than 10% of breast tumors are missed by mammography, leading to a sensitivity of 70% to 91% [5]. In contrast, MRI requires no breast compression and offers excellent imaging around dense breast tissue. Unfortunately, the high sensitivity (85% to 100%) of MRI is compromised by a high rate of false positives (37% to 100%), which call for unnecessary follow-up examinations and invasive biopsies [6]. Regarding these limitations of mammography and MRI in population-based screening, minimally invasive screening tests are desirable that could complement mammography or MRI, or even stand alone as a primary screening tool. Confidence is growing that the next generation of screening tests will be based on molecular biomarkers present in bodily fluids.

Determination of promoter methylation of tumor-suppressor genes in the DNA of easily accessible bodily fluids, like serum or plasma, is a rapidly growing research field in cancer detection. The principle of DNA methylation markers for early breast cancer detection is based on evidence that growing tumors release significant amounts of circulating free DNA (cfDNA) into the bloodstream through cellular necrosis, apoptosis, or spontaneous release of DNA into the circulation from primary and metastatic tumors [7,8]. The diagnostic potential of *RASSF1A *and *APC *promoter methylation in cfDNA from breast cancer patients has been investigated in several studies [9-12]. In particular, *RASSF1A *methylation of cfDNA predicted the presence of breast cancer with a sensitivity ranging from 15% to 75%, whereas with *APC*, methylation sensitivities from 2% to 47% were achieved. For either, gene specificity in healthy controls was claimed to be high (90% to 100%).

Although several potential DNA methylation biomarkers have been reported, none of these has reached clinical practice. A major limitation to further development for clinical use might have been that these studies analyzed small numbers of breast cancers and matched control specimens, and validations with larger patient cohorts were not pursued [13-15]. Furthermore, investigations of the methylation patterns in cfDNA from non-breast cancers or different benign breast diseases to identify potential specificity issues is lacking in most of these studies. Colon cancer in women is by 60% less frequent than breast cancer [1] (for example, *APC *methylation is also detected in cfDNA from colon cancer patients with considerable sensitivity) [16]. Therefore, supposed specific biomarkers are reduced in their overall specificity when analyzing additional serum samples from non-breast tumor patients. In addition, fibroadenoma tumors, the most common benign tumors in women, typically found during the middle and later reproductive years, are a potential source for hypermethylated cfDNA [17], indicating that high specificity in benign disease controls is mandatory to establish a suitable biomarker panel for early breast cancer detection.

In the present study, seven potential breast cancer marker candidates (*SFRP1, SFRP2, SFRP5, WIF1, DKK3, ITIH5*, and *RASSF1A*) were studied with regard to early breast cancer detection in serum. These genes have previously shown cancer-specific methylation in breast tissue [18-22]. So far, no studies have considered the promoter methylation of these genes in a large set of breast cancer serum samples, as well as different specificity controls. We examined promoter methylation of these candidate genes in two independent sets with a total of 604 serum samples and 112 matched breast cancer tissues. In a test set (*n *= 261 sera; *n *= 112 tissues), we determined the best candidate markers with the highest specificity in age-matched healthy and benign disease controls. An independent validation set (*n *= 343 sera) was then used to validate the top genes obtained from the test study as reliable detection markers.

## Materials and methods

### Patients

**Figure 1:**
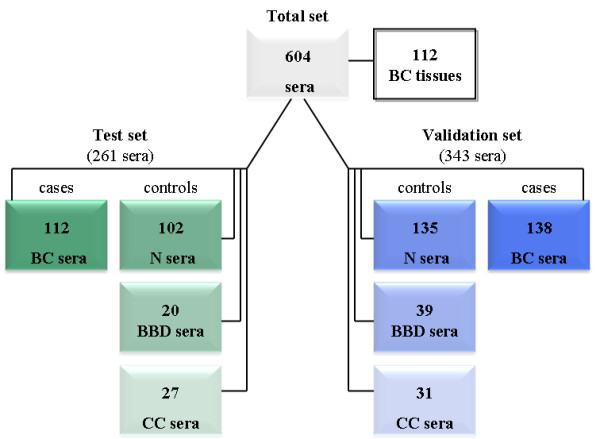
**Overview of the analyzed serum samples in two independent sets and paired breast cancer tumor tissues**. BBD, benign breast disease; BC, breast cancer; CC, colon cancer;N, healthy normal.

In total, 604 serum samples were assessed in this case-control study. It included samples from patients with all stages of breast cancer (*n *= 250 sera), cancer-free individuals (*n *= 237 sera), patients with benign breast disease (*n *= 59 sera), and patients with colon cancer (*n *= 58 sera). Importantly, 90% of the patients in both the test and the validation set had small tumors (pT1 or pT2). Furthermore, asymptomatic women who, based on their family history and/or mutational analysis, were suspected or proven to carry mutations in breast cancer susceptibility genes (*BRCA1 *and *BRCA2*) or had a previous breast tumor were not included in our group of healthy controls. An overview of the analyzed serum sets is summarized in Figure 1. A subset of 261 serum and 112 paired breast cancer tissue samples was used as test set, and was obtained from the tumor bank of Euregional comprehensive Cancer Center Aachen (ECCA), now part of the RWTH centralized biomaterial bank (RWTH cBMB). All patients gave informed consent for retention and analysis of their serum for research purposes (local ethical review board of the medical faculty of the RWTH Aachen, ref no. EK-206/09). The validation set consisted of 343 samples, including clinicopathologically matched samples to the test set. Samples of the validation set were obtained from the University Hospital of Erlangen and from Patients' Tumor Bank of Hope (PATH foundation, a research resource for breast cancer biosamples) [23]. Additional controls were collected from individuals with colon cancer, because colon cancer is the second common cancer type in women. In addition, various sera from benign breast disease patients, including fibroadenoma (*n *= 17), desmoid tumors (*n *= 1), benign phyllodes tumors (*n *= 1), mastopathy (*n *= 33), papilloma (*n *= 5), duct ectasia (*n *= 1), and harmatoma (*n *= 1) were analyzed for additional specificity testing. Additional control samples were obtained from the RWTH cBMB. An overview of the clinical characteristics of the breast cancer patients in the test and validation set is summarized in Table 1. All subjects participating were HIV, HBV, and HCV negative and had no previous history of cancer. Blood from all patients was drawn immediately or up to 2 days after diagnosis and before starting any cancer-specific treatment. All patient samples were collected between the years 2005 and 2012.

**Table 1:** Clinicopathologic parameters of patients with breast cancer in the test and validation sets

Categorization	Test set (*n*^a ^= 112)	%	Validation set (*n*^a ^= 138)	%	*P *value^b^
Age at diagnosis					0.327
	
Median	60 years (range, 36-87 years)		63 years (range, 33-86 years)		

Menopausal status					0.791
	
Premenopausal	22	19.6	21	15.2	
	
Postmenopausal	90	80.4	117	84.8	

Tumor size^c^					0.337
	
pT1	49	43.8	67	48.6	
pT2	51	45.5	59	42.8	
pT3	8	7.1	8	5.9	
pT4	2	1.8	2	1.4	
Unknown	2	1.8	2	1.4	

Lymph node status^c^					0.997
	
pN0	55	49.1	77	55.8	
pN1	38	33.9	42	30.4	
pN2	9	8.0	10	7.2	
pN3	7	6.3	6	4.3	
Unknown	3	2.7	3	2.2	

Histologic type					0.617
	
Invasive ductal	89	79.5	115	83.3	
Invasive lobular	15	13.4	17	12.3	
Other	8	7.2	6	4.2	

Tumor grade^d^					0.175
	
G1	6	5.4	14	10.1	
G2	62	55.4	76	55.1	
G3	44	39.3	48	34.8	

Estrogen-receptor status^e^					0.293
	
Negative (IRS0-2)	22	19.7	27	19.5	
Positive (IRS3-12)	84	75.0	109	78.9	
Unknown	6	5.4	2	1.4	

Progesterone-receptor status^e^					0.112
	
Negative (IRS0-2)	30	26.8	39	28.2	
Positive (IRS3-12)	76	68.0	96	69.3	
Unknown	6	5.4	3	2.2	

HER2 status^f^					0.112
	
Negative (0; 1+; 2+)	90	80.4	114	82.6	
Positive (3+)	14	12.5	20	14.9	
Unknown	8	7.1	4	2.9	

^a^Only female patients with primary, unilateral, invasive breast cancer were included. ^b^Fisher Exact test at a two-sided significance level of 0.05. ^c^According to TNM classification by Sobin and Wittekind [41]. ^d^According to Bloom and Richardson, as modified by Elston and Ellis [42]. ^e^IRS, immunoreactive score according to Remmele and Stegner [43]. ^f^Overexpression for the *HER2/neu *gene was diagnosed analogous to the threshold of the DAKO-Score system based on IHC assay. Percentages may not sum to 100% because of rounding.

Tumor material was snap-frozen in liquid nitrogen directly after surgery. Hematoxylin and eosin-stained sections were prepared for assessment of the percentage of tumor cells, but only samples with > 70% tumor cells were selected. Blood samples (10 ml) from all study participants were obtained by venipuncture. Samples were immediately centrifuged at 2,000 *g *for 10 minutes at room temperature, and 1-ml serum aliquots were stored at -80°C until use.

### Nucleic acid extraction

Genomic DNA from breast tumors was isolated by using the QIAamp DNA Mini Kit (Qiagen, Hilden, Germany) according to the manufacturers' recommendations.

CfDNA was extracted from 1 ml serum by using the QIAamp Circulating Nucleic Acid Kit (Qiagen) according to the manufacturer's recommendations, eluted in 35 μl of TE buffer, and quantified spectrophotometrically. In the test set, the median quantity of cfDNA in breast cancer serum was 903 ng DNA/ml serum (range, 304.5 to 8,291.5) and 889 ng DNA/ml serum (range, 297.5 to 1,893.5) in healthy controls. In the validation set, the median quantity of 791 ng DNA/ml serum (range, 164.5 to 1,680) cfDNA was obtained from breast cancer sera, and 833 ng DNA/ml (range, 332.5 to 3,010) from healthy controls. DNA samples were stored at -80°C until use.

### DNA bisulfite modification

The extracted tissue DNA and serum cfDNA was bisulfite-converted by using the EZ DNA methylation kit (Zymo Research, Orange, CA, USA) as previously described [18].

### Methylation-specific polymerase chain reaction (MSP)

The methylation status of the different genes was determined qualitatively by methylation- specific polymerase chain reaction (MSP) [24] and quantitatively by MethyLight technology [25]. For MSP, bisulfite-converted DNA was amplified by using primer sets specific for unmethylated and methylated promoter sequences in each candidate gene. PCRs were run in a volume of 25 μl, containing 20 ng bisulfite-modified DNA, 2.5 μl 10 × MSP PCR buffer [26], 1.25 μl dNTPs (25 m*M*), 0.5 μl sense primer (10 μ*M*), 0.5 μl antisense primer (10 μ*M*), 1.25 units GoTaq Flexi DNA polymerase (Promega, Madison, WI, USA) (0.25 μl Taq + 4.75 μl H_2_O), and 14.25 μl H_2_O. The PCR profile was 95°C for 5 minutes, 38 to 40 cycles at 95°C for 30 seconds, primer annealing at 59°C to 63°C for 30 seconds, 72°C for 30 seconds, and a final extension step at 72°C for 10 minutes. MSP primer sequences and annealing temperatures are listed in Additional file 1. PCR amplificates were electrophoresed on 3% agarose gels and evaluated under ultraviolet light. Universal bisulfite-converted polymethylated and unmethylated DNA (Epi Tect Control DNA Set, Qiagen) served as positive controls, and H_2_O and genomic unmodified DNA were included as negative controls in each run.

### Quantitative methylation-specific polymerase chain reaction (qMSP)

Bisulfite-modified DNA was used as template for fluorescence-based real-time PCR, as previously described [25]. Amplification reactions were carried out in triplicate in a volume of 25 μl containing 20 ng bisulfite-modified serum DNA, 12.5 μl IQ Supermix (Biorad, Munich, Germany), 0.75 μl sense primer (10 μ*M*), 0.75 μl antisense primer (10 μ*M*), 0.5 μl probe (10 μ*M*), and 10.5 μl H_2_O. Primers and probes were designed specifically to amplify bisulfite-converted DNA for the genes of interest (*DKK3*, *ITIH5*, and *RASSF1A) *and the reference housekeeping gene (*GAPDH) *to normalize for input DNA. Amplifications were carried out by using the following profile: 95°C for 10 minutes, 40 to 50 cycles at 95°C for 15 seconds, and primer annealing at 58°C to 66°C for 30 seconds. QMSP primer sequences and annealing temperatures are listed in Additional file 2.

Amplifications were performed in an iCycler iQ5 (Bio-Rad). Each plate included positive controls for the methylated sequence (*in vitro *methylated human leukocyte DNA) and unmethylated sequence (human leukocyte DNA from a healthy donor), as well as multiple water blanks. Primer binding sites of the qMSP assays were located in the same genomic promoter region as primers used for MSP analyses.

### Analytic performance of the blood-based qMSP assays

Leukocyte DNA from healthy individuals was methylated with *SssI *(CpG) methyltransferase (New England Biolabs, Beverly, MA, USA) to generate completely methylated DNA. Serial dilutions (500 ng to 0.025 ng) of this DNA were used to construct a calibration curve for each plate. Control DNA for serial dilutions was diluted before bisulfite conversion. All analyzed samples were within the different assays range of sensitivity and reproducibility, based on amplification of internal reference standard (cycle threshold (C_T_) value for *GAPDH *of 32 or less). The gene of interest was called methylated if the C_T _of at least two of three PCR replicates for each specimen had a value of less than 45 cycles. Genes of interest were considered unmethylated if C_T _was not measurable or was ≥ 45.0 and the *GAPDH *C_T _was ≥ 33 cycles. The amount of methylated DNA (percentage of methylated reference, PMR) at a specific locus was calculated by dividing the *GENE/GAPDH *ratio of a sample by the *GENE/GAPDH *ratio of *Sss*I-treated human leukocyte DNA and multiplying by 100 [25]. Additionally, analytic performance of the *DKK3*, *ITIH5*, and *RASSF1A *assay was determined by using the *in vitro *methylated human leukocyte DNA added in definite concentrations (500 ng to 0.01 ng) to 1 ml disease-free human serum. Next, the added methylated DNA was isolated and bisulfite-treated as mentioned earlier. The detection limit of *DKK3 *and *RASSF1A *methylation at the specimen level was 50 pg/ml, and of *ITIH5 *methylation, 100 pg/ml.

### Statistical analysis

Statistical analyses were performed by using SPSS 17.0 (SPSS, Chicago, IL, USA) and GraphPad Prism 5.0 (GraphPad Software Inc., La Jolla, CA, USA). *P *values of < 0.05 were considered significant. Two-sided Fisher Exact tests were performed to compare clinicopathologic factors between cohorts, and to correlate clinicopathologic parameters with the methylation values of the candidate genes in the test and validation sets. A Mann-Whitney *U *test was used to detect significantly differential methylation levels between serum of age-matched healthy women, benign breast diseases, sera from colon cancers, and breast cancer. Mean methylation values of the three-gene panel were visually compared by using scatterplots of the log-transformed PMR values. Receiver-operating-characteristics (ROC) curves were calculated to evaluate the diagnostic performance of different marker combinations [27].

## Results

### Determination of the best-performing genes for a breast cancer blood test with qualitative MSP

We initially assessed promoter methylation of *SFRP1*, *SFRP2*, *SFRP5*, *ITIH5*, *DKK3*, and *WIF1 *in 112 paired breast cancer tissue and serum samples by using qualitative MSP. The majority of genes methylated in cfDNA were also methylated in tissue DNA, whereas methylation of tissue DNA was less frequently retrieved in cfDNA. A significant positive correlation between promoter methylation of *DKK3 *(*P *= 0.003) and *ITIH5 *(*P *= 0.007) in paired tissue DNA and serum cfDNA was found (see Additional file 3). *SFRP2 *was methylated in four cases, and both *SFRP1 *and *ITIH5 *in two cases of cfDNA, with no corresponding methylation in the paired-tissue DNA. In general, methylation frequencies of *ITIH5*, *WIF1*, and *DKK3 *in cfDNA were at least 36% compared with that of tissue DNA, whereas *SFRP1*, *SFRP2*, and *SFRP5 *exhibited just 9% to 24% of cfDNA methylation compared with the methylation frequency in paired tissue DNA (Table 2).

**Table 2:** Detection of aberrant gene promoter methylation in paired samples (serum and tumor) in the test set

	Sensitivity in breast cancer serum		Sensitivity in breast cancer tissue	
**Gene**	**Methylation** **Positive**	**%**	**Methylation** **positive**	**%**

*ITIH5*	27 of 112	**24**	81 of 112	**72**
*DKK3*	37 of 112	**33**	97 of 112	**87**
*WIF1*	39 of 112	**35**	108 of 112	**96**
*SFRP1*	12 of 112	**11**	89 of 111	**79**
*SFRP2*	21 of 112	**19**	90 of 112	**80**
*SFRP5*	9 of 112	**8**	104 of 110	**93**
*DKK3/ITIH5^a^*	46 of 112	**41**	105 of 112	**94**

^a^Either gene methylated.

Next, we investigated methylation of the six candidate genes in 102 age-matched healthy controls and 47 additional disease controls. An overview of the methylation frequencies in the studied serum samples of the test set is given in Tables 2 and 3. Based on the methylation frequencies, we defined for the most suitable genes for a clinical assay the following criteria: methylated in ≤ 7% of serum samples from healthy controls and benign disease controls, ≤ 20% in colon cancer serum samples, and ≥ 20% in serum samples from breast cancer patients. On the basis of the defined criteria, *SFRP5 *methylation (8.25%) was too infrequent in breast cancer sera, and thus excluded from further specificity analyses. Similarly, *SFRP1 *and *SFRP2 *showed high methylation rates in healthy controls and benign disease samples (Table 3), and thus were excluded.

**Table 3:** Sensitivity and specificity of serum-based detection of aberrantly methylated genes in the test set

	Sensitivity in breast cancer		Specificity in healthy controls		Specificity in benign breast diseases	
**Gene**	**Methylation** **positive**	**%**	**Methylation** **negative**	**%**	**Methylation** **negative**	**%**

*ITIH5*	27 of 112	**24**	96 of 102	**94**	20 of 20	**100**
*DKK3*	37 of 112	**33**	101 of 102	**99**	20 of 20	**100**
*WIF1*	39 of 112	**35**	97 of 102	**95**	15 of 20	**75**
*SFRP1*	12 of 112	**11**	92 of 102	**90**	17 of 20	**85**
*SFRP2*	21 of 112	**19**	90 of 102	**88**	18 of 20	**90**
*SFRP5*	9 of 112	**8**	n.d.		n.d.	
*DKK3/ITIH5*^a^	46 of 112	**41**	95 of 102	**93**	20 of 20	**100**

^a^Either gene methylated. n.d., not done.

Finally, *WIF1 *was excluded because this gene exhibited an abundant methylation rate (25%) in serum samples from benign breast diseases. Additional specificity testing in 27 colon cancer samples revealed moderate methylation frequencies in all candidate markers with the exception of *DKK3 *(Table 4). Two genes, *ITIH5 *and *DKK3*, met our defined criteria, and were highly specifically unmethylated in sera from healthy women and those affected with benign disease, as well as sera from non-breast tumors. In addition, only *DKK3 *and *ITIH5 *methylation showed a significant concordance in tumor tissue and paired serum specimens. The combination of *DKK3 *and *ITIH5 *(either gene methylated) substantially improved sensitivity and specificity, which predicts breast cancer with a sensitivity of 41% and a specificity of 93%, 100%, and 88% in age-matched healthy control samples, benign breast disease, and colon cancer samples, respectively. Considering all control samples, including sera from patients with colon cancer, benign breast disease, and healthy controls, yielded a specificity of 93% (139 of 149) with combined *DKK3 *and *ITIH5 *methylation.

**Table 4:** Additional specificity testing of aberrantly methylated genes in colon cancer in the test and validation sets

	Specificity in colon cancer			
	
	Test set		Validation set	
**Gene**	**Methylation** **negative**	**%**	**Methylation** **negative**	**%**

*DKK3*	27 of 27	**100**	24 of 31	**77**
*ITIH5*	24 of 27	**89**	22 of 31	**71**
*WIF1*	23 of 27	**85**	n.d.	
*SFRP1*	16 of 22	**73**	n.d.	
*SFRP2*	22 of 26	**85**	n.d.	
*SFRP5*	n.d.		n.d.	
*RASSF1A*	n.d.		13 of 31	**42**
*DKK3/ITIH5*^a^	24 of 27	**88**	16 of 31	**52**
Three-gene panel^a^	n.d.		6 of 31	**19**

^a^Either gene methylated. n.d., not done.

### Detection of breast cancer in an independent validation set by using *ITIH5, DKK3*, and *RASSF1A *

To confirm the clinical performance of *DKK3 *and *ITIH5 *methylation, we used serum samples from an independent patient set. In the validation data analysis, 138 breast cancer samples, 135 samples from age-matched healthy controls, and 70 additional disease controls were analyzed. To increase the analytic sensitivity and to yield a higher reproducibility [28,29] we analyzed the validation set with qMSP. Primer binding sites of both the MSP and the qMSP assays were located in the same genomic promoter region but did not cover identical CpG dinucleotides. Nevertheless, methylation frequencies of the analyzed genes in the test and the validation sets were very highly similar, independent of the technology applied. To study the correlation between methylation obtained with MSP and qMSP, serum samples of breast cancer patients of the validation set, including negative and positive cases, were reexamined with qualitative MSP. A significant positive correlation for both *ITIH5 *(*P *= 0.040) and *DKK3 *(*P *= 0.033) promoter methylation between both technologies was found.

Methylation of *DKK3 *was present in serum from two (1.5%) of 135 matched control subjects and 41 (29.7%) of 138 breast cancer patients (*P *< 0.0001). In addition, specificity in benign disease controls was high (all 39 (100%) samples were unmethylated). *ITIH5 *was methylated in seven (5.2%) of 135 healthy controls, in one (2.6%) of 39 benign controls, and 19 (13.8%) of 138 breast cancer samples. The combination of *DKK3 *and *ITIH5 *methylation revealed a sensitivity of 39.9% (55 of 138) for the detection of breast cancer and a specificity of 93.3% (126 of 135) and of 97.4% (38 of 39) in healthy controls and benign disease controls (*P *< 0.0001), respectively. In serum samples from patients with colon cancer, *DKK3 *was detected in seven (22.6%) of 31, whereas *ITIH5 *was present in nine (29%) of 31 cases (Table 4).

**Figure 2:**
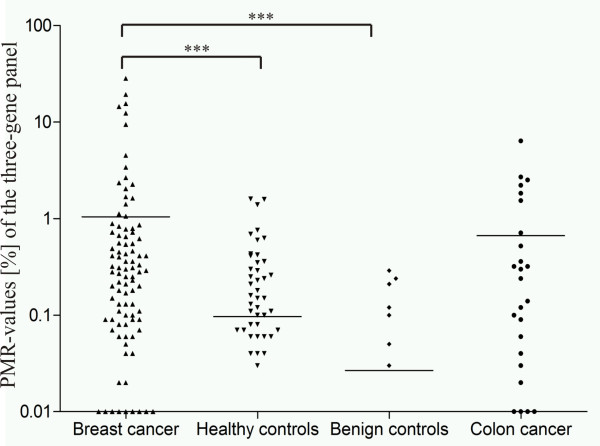
**The biomarker panel including *ITIH5*, *DKK3*, and *RASSF1A *enables significant discrimination of breast cancer sera from various control conditions**. Scatterplot illustrating the mean PMR values of the three-gene panel in the independent validation set. Combination of *DKK3 *and *ITIH5 *with *RASSF1A *increased the sensitivity to 67% (92 of 138) with a specificity of 69% (93 of 135) and 82% (32 of 39) in healthy and benign disease controls, respectively. ***P < 0.0001.

To improve the sensitivity of the panel, we combined *DKK3 *and *ITIH5 *with the potential biomarker *RASSF1A*. *RASSF1A *methylation was significantly different between serum of breast cancer patients (64 (47.1%) of 136), and both healthy controls (35 (25.9%) of 135) (*P *= 0.0035) and benign disease controls (six (15.4%) of 39; *P *= 0.0013). The examination of 31 additional samples from patients with colon cancer indicated low specificity of *RASSF1A *methylation (42%) in cfDNA (Table 4). Figure 2 illustrates the distribution of mean PMR values for the three-gene panel with *ITIH5*, *DKK3*, and *RASSF1A*. A significant (*P *= 0.040) positive correlation of the methylation status also was obtained with MSP and qMSP in 12 samples (six negative and seven positive cases) of the validation set.

**Figure 3:**
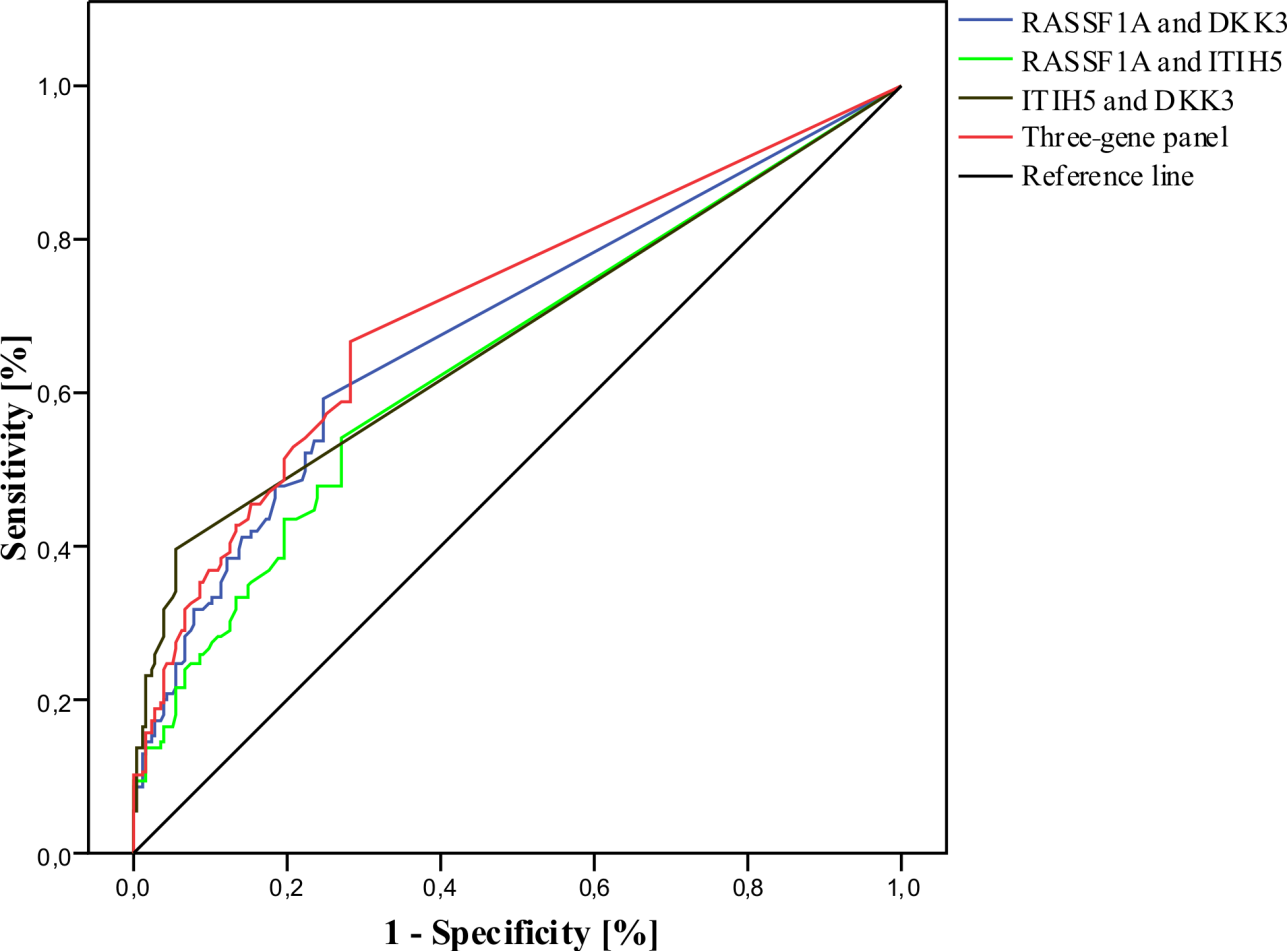
**ROC-curve analysis of the three-gene panel in control samples including age-matched healthy and benign disease controls**. ROC curves were established for different biomarker combinations to determine a cutoff value. A cutoff value of 0.085% methylation was defined for positive detection of disease; the specificity of the panel increased to 80.5% with a sensitivity of 51.4%. ROC analysis revealed an area under the curve (AUC) of 0.712 (95% CI, 0.653 to 0.770).

Next, we performed ROC curve analysis for multiple marker combinations to determine both sensitivity and specificity in breast cancer and control samples and to calculate the optimal threshold value for the three-marker panel (Figure 3). According to this analysis, the three-marker panel can discriminate between breast cancer patients and women without breast cancer with a sensitivity of 67% and a specificity of 69% (AUC, 0.697 (95% CI, 0.634 to 0.759)). Additionally, the three-marker panel allowed distinguishing breast cancer patients from women with a benign breast disease with high specificity (82%; AUC, 0.765 (95% CI, 0.692 to 0.839)). The combination of healthy and benign controls revealed a specificity of the three-marker panel of 72% (AUC, 0.712 (95% CI, 0.653 to 0.770)). Combinations of different markers were examined to maximize specificity and sensitivity. The best combination remained with all three genes (Table 5). Defining a cut-off value for the three-gene panel of 0.085% for positive detection of methylation (and thus disease), the panel distinguished between breast cancer and both healthy and benign controls with a sensitivity of 51.4% and a specificity of 80.5%.

**Table 5:** Sensitivity and specificity of breast cancer detection with different marker combinations

	Sensitivity in breast cancer		Specificity in healthy and benign breast disease controls		*P *value^b^	AUC (95% CI)	PPV	NPV
				
Gene^a^	Methylation positive	%	Methylation negative	%			%	%
*RASSF1A* *ITIH5*	75 of 138	**54**	127 of 174	**73**	7.8 × e^-6^	0.647 (0.585-0.709)	61	67

*RASSF1A* *DKK3*	82 of 138	**59**	131 of 174	**75**	1.6 × e^-8^	0.686 (0.626-0.747)	66	70

*DKK3* *ITIH5*	55 of 138	**40**	164 of 174	**94**	1.6 × e^-7^	0.673 (0.611-0.735)	85	66

*RASSF1A* *DKK3* *ITIH5*	**92 of 138**	**67**	**125 of 174**	**72**	**1.3 × e^-10^**	**0.712 (0.653-0.770)**	65	73

^a^Either gene methylated. ^b^Fisher Exact test at a two-sided significance level of 0.05. AUC, area under the curve; CI, confidence interval; NPV, negative predictive value; PPV, positive predictive value.

### Correlation of *ITIH5*, *DKK3*, and *RASSF1A *methylation with clinicopathologic parameters

The methylation status of *ITIH5*, *DKK3*, and *RASSF1A *in breast cancer tissue DNA and serum cfDNA in both the test and validation sets was analyzed for association with known clinicopathologic characteristics, including age at diagnosis, tumor grade, tumor size, node status, hormone-receptor status, and menopausal status. Detection of *ITIH5*, *DKK3*, and *RASSF1A *methylation in the test and the validation sets was independent of patient age (*P *> 0.05). However, in the test set, sensitivity of *DKK3 *and *ITIH5 *methylation and sensitivity of the combined methylation of *DKK3*/*ITIH5 *was more frequent in premenopausal women. In the validation set, methylation frequency of either *DKK3*, or methylation of the gene combination *DKK3*/*ITIH5 *or methylation of the three-gene panel was also increased in premenopausal women, whereas the methylation frequency of *RASSF1A *was more frequent in the postmenopausal subgroup. Area under the curve (AUC) values were improved in premenopausal women (see Additional file 4). Furthermore, statistical investigations of the validation set revealed a significant association of *DKK3 *cfDNA methylation (*P *= 0.034) and cfDNA methylation of the *DKK3 *and *ITIH5 *gene combination (*P *= 0.007) with smaller tumors (pT1-2).

## Discussion

In this study, we determined the performance of a blood-based PCR assay for methylated cfDNA of seven potential biomarkers in two independent serum sets with a total of 604 serum and 112 paired tumor tissue samples. Previous studies showed the involvement of the candidate genes in signal transduction of the WNT pathway (*SFRP1*, *SFRP2*, *SFRP5*, *DKK3*, and *WIF1*) [19,20,22], invasion and metastasis (*ITIH5*) (18), and regulation of apoptotic and cell-cycle checkpoint pathways (*RASSF1A*) [30], suggesting a role in breast carcinogenesis. The potential use of these genes, except *RASSF1A*, as blood-based biomarkers for breast cancer was not investigated previously. However, the high methylation frequencies previously described for these genes in breast tumor tissue suggested their potential feasibility for early breast cancer detection in cfDNA. This is the first study aiming to develop a blood-borne PCR assay to detect breast cancer by using two independent series of breast cancer serum samples, thus composing the largest sample collection analyzed for serum-based cfDNA methylation markers.

Methylation changes in carcinogenesis are often heterogeneous, and still no single gene has been found to be methylated in every breast cancer specimen. Furthermore, in most studies investigating methylation levels of only single genes, the sensitivity was low [31]. Therefore, it is considered advantageous to use a panel of genes for breast cancer screening procedures. Recent studies have reported a range of potential gene panels, including the most frequently methylated genes in human breast cancer [11,12,32-34]. In agreement with Van de Voorde *et al. *[31], most of these potential biomarkers have been analyzed in relatively small patient cohorts, and further validation in larger-scaled patient and age-matched healthy cohorts was not pursued. Furthermore, the frequent lack of ROC analyses in most biomarker studies may result in misinterpretation of the analyzed data [31]. Because of the comparative analysis of 112 paired breast cancer tumor tissues and serum samples, as well as the investigation of a strong and realistic cohort of control samples in our study, we were able to define a biomarker panel with the best values for breast cancer specificity first in a test set. *ITIH5 *and *DKK3 *proved to be candidate genes showing a significant correlation of the methylation pattern in paired tumor tissue DNA and cfDNA. Concordance of the methylation pattern found in tissue and serum specimens indicates the potential utility of blood-based detection of breast cancer. Furthermore, *ITIH5 *and *DKK3 *methylation achieved 40% sensitivity with a high specificity in healthy and benign disease controls. In an independent validation set, *DKK3 *and *ITIH5 *were further validated in combination with the known biomarker *RASSF1A *to increase sensitivity of the panel. Indeed, the combination of *DKK3 *and *ITIH5 *with *RASSF1A *increased sensitivity from 40% to 67%, but specificity of *RASSF1A *in healthy controls was quite low (100 (74%) of 135), resulting in a reduction of specificity from 93% to 69% with the three-marker panel. The combination of healthy and benign controls increased specificity of the three-gene panel to 72%. Recent publications reported *RASSF1A *methylation frequencies of 32% to 75% in cfDNA of breast cancer patients, in line with our data from the validation set (sensitivity of 46%) [30]. In particular, Brooks *et al. *[12] investigated *RASSF1A *promoter methylation in 50 breast cancer serum samples, 100 healthy, and 50 benign breast disease controls. Sensitivity of *RASSF1A *was much lower (22%) than in our study, whereas specificity in healthy controls was slightly higher. However, specificity of *RASSF1A *was lower in our study compared with that in other publications [10,34]. This may be due to the examination of a larger set of age-matched healthy control samples compared with these studies. The potential biomarkers *DKK3 *and *ITIH5 *seem to be more breast cancer specific (94% to 99%) but less sensitive (14% to 33%) in both the test and the validation sets. Nevertheless, *DKK3 *promoter methylation could exhibit the feasibility to detect cfDNA methylation of smaller tumors (pT1-2), which also remains valid in combination with *ITIH5*. ROC analysis of cfDNA methylation of pT1-2 tumors increased the sensitivity of the *DKK3 *and *ITIH5 *gene combination in the validation set from 40% to 43% (AUC, 0.688 (95% CI, 0.624 to 0.752)), which might indicate a potential benefit of *DKK3 *and *ITIH5 *for the early detection of breast cancer. However, the validation set comprised only 9% pT3-4 tumors, so further studies are needed. Additionally, *DKK3 *and *ITIH5 *specificity in colon cancer cfDNA is much higher in comparison to *RASSF1A*, indicating the eligibility of *DKK3 *and *ITIH5 *as potential breast cancer biomarkers.

Regarding mammography, with a sensitivity of 70% and a specificity of > 85%, a reliable biomarker panel for the early detection of breast cancer in cfDNA has to accomplish comparable values. However, sensitivity of mammography declines drastically in patients with dense breast tissue [35], and additionally, mammographic density is a strong risk factor for breast cancer [36-38]. We hypothesize that *DKK3 *and *ITIH5 *may be valuable biomarkers for the blood-based detection of breast cancer in patients with dense breast tissue because of their high specificity in the control specimens. Prospectively, *DKK3 *and *ITIH5 *methylation analyses may be used in supplement to mammography, the sensitivity of which is low in patients with dense breast tissue. With ROC curve analysis, we examined *DKK3*, *ITIH5*, and also *RASSF1A *methylation with regard to sensitivity and specificity in pre- and postmenopausal women. Interestingly, in the validation set, methylation of either *DKK3*, or methylation of the *DKK3*/*ITIH5 *gene combination or methylation of the three-gene panel is more frequent in premenopausal women, whereas *RASSF1A *methylation is increased in postmenopausal women. In the validation set, ROC analysis of cfDNA methylation of premenopausal women revealed a higher sensitivity (52%) of the *DKK3 *and *ITIH5 *gene combination with a specificity of 100% (AUC, 0.762 (95% CI, 0.611 to 0.913)), whereas the *DKK3 *and *ITIH5 *gene combination exhibited a sensitivity of 36% with a specificity of 92% (AUC, 0.647 (95% CI, 0.575 to 0.718)) in postmenopausal women. These results might indicate a pronounced benefit of *DKK3 *and *ITIH5 *for the early detection of breast cancer in premenopausal women. However, the premenopausal subgroup in both test and validation sets comprised only 15% to 20% of cases, so further studies are needed to confirm this finding.

In summary, the three-marker panel with *ITIH5*, *DKK3*, and *RASSF1A *exhibits significantly increased levels of methylation in serum cfDNA from breast cancer patients compared with both age-matched healthy women and women with a benign breast disease. According to our data, *RASSF1A *promoter methylation is not an ideal biomarker for early breast cancer detection because it shows a high frequency of methylation in cfDNA derived from sera of healthy individuals. However, although *DKK3 *and *ITIH5 *are highly specific in healthy and benign control samples, sensitivity of the potential biomarkers is limited.

Our next goal is to discover and characterize more potential biomarkers for early breast cancer detection with a quality comparable to that of *ITIH5 *and *DKK3 *promoter methylation. In addition, other approaches like investigations of copy-number variations (CNVs), loss of heterozygosity (LOH), or point mutations in cfDNA could be used to improve the sensitivity of the two-gene combination. A study by Shaw *et al. *[39] investigated CNVs and LOH in cfDNA of breast cancer patients and matched controls and demonstrated different potential gene targets that distinguish between patients with primary breast cancer and healthy controls. It is conceivable that *DKK3 *and *ITIH5 *sensitivity could be improved through the additional investigation of CNVs of genes like, for example, *MAT1 *or *JAG2*. Another way to improve our approach could be the additional analysis of gene mutations in cfDNA (such as *PIK3CA *or *TP53*). Board *et al. *[40] investigated the potential utility of cfDNA as a source for *PIK3CA *mutation detection in patients with breast cancer. *PIK3CA *mutations were detected in 13 (28%) of 46 plasma-derived and 10 (21%) of 46 serum-derived cfDNA samples from metastatic breast cancer patients. Interestingly, no *PIK3CA *mutations were detected in cfDNA from localized breast cancers (*n *= 30). Altogether, this could allow establishing a biomarker panel offering a sensitivity and specificity comparable to that of mammography examination. Of importance, such a blood-borne screening test would be more convenient for the patient and less expensive for the health-care system.

## Conclusions

Quantifying promoter methylation of putative tumor-suppressor genes in circulating free DNA (cfDNA) of bodily fluids, like serum, is a rapidly growing research topic for early cancer detection. However, in the breast cancer field, none of the reported biomarkers has reached clinical application, mainly because of the small numbers of breast cancer and matched control specimens analyzed. In the current study, seven potential biomarkers were evaluated in two independent serum sets (*n *= 604). Promoter methylation of the *DKK3*/*ITIH5 *gene combination allowed significant discrimination of breast cancer sera from various control conditions. Moreover, these biomarkers showed the feasibility of detecting cfDNA methylation even in smaller tumors and in premenopausal women. A simple and noninvasive blood-borne screening test could complement image-driven screening technologies like mammography or magnetic resonance imaging (MRI) in early breast cancer detection.

## Abbreviations

*APC*: adenomatous polyposis coli; AUC: area under the curve; cBMB: centralized biomaterial bank; cfDNA: circulating-free DNA; C_T_: cycle threshold; DCIS: ductal carcinoma *in situ*; DNA: deoxyribonucleic acid; *DKK3*: Dickkopf 3; *GAPDH*: glyceraldehyde-3-phosphate dehydrogenase; HBV: human hepatitis B virus; HCV: human hepatitis C virus; HIV: human immunodeficiency virus; *ITIH5*: inter-alpha-trypsin inhibitor chain 5; MSP: methylation-specific PCR; PATH: Patients' Tumor Bank of Hope; PCR: polymerase chain reaction; PMR: percentage of methylated reference; *RASSF1A*: Ras association domain family member 1A; ROC: receiver operating characteristic; *SFRP1*: secreted frizzled-related protein 1; *SFRP2*: secreted frizzled-related protein 2; *SFRP5*: secreted frizzled-related protein 5; *WIF1*: Wnt inhibitory factor 1.

## Competing interests

The authors have declared that no competing interests exist.

## Authors' contributions

VK, RK, and ED participated in the design of the study. VK, BB, KW, MGS, PAF, TA, JV, and AH carried out the experimental data acquisition. VK, BB, MGS, PAF, JV, and ED performed data analyses. VK wrote the manuscript. All authors read, critically revised, and approved the final manuscript.

## Supplementary Material

Additional file 1 **Sequences for the MSP primer and performing conditions**. The table shows the primer sequences of the genes analyzed with MSP, the performing conditions, and the product size. Click here for file

Additional file 2 **Sequences for the MethyLight primer and performing conditions**. The table shows the primer sequences of the genes analyzed with qMSP, the performing conditions, and the product size. Click here for file

Additional file 3 **Correlation of the methylation level in tissue DNA and cfDNA in paired breast cancer samples**. This table provides the correlation and *P *values between *DKK3 *and *ITIH5 *promoter methylation in paired breast cancer tissue DNA and serum cfDNA. Click here for file

Additional file 4 **Sensitivity and specificity of breast cancer detection in pre- and postmenopausal women in the test and validation sets**. This table provides sensitivity and specificity of *ITIH5*, *DKK3*, and *RASSF1A *methylation in pre- and postmenopausal women in the test and validation sets. In addition, area under the curve (AUC) values for all biomarker candidates are shown in these two strata. Click here for file
