# Supplementary material for: Promoter hypermethylation of the tumor-suppressor genes ITIH5, DKK3, and RASSF1A as novel biomarkers for blood-based breast cancer screening
Source: Breast Cancer Res. 2013 Jan 15;15(1):R4. doi: 10.1186/bcr3375 (PMC3672828; doi:10.1186/bcr3375)
Supplement: Additional file 1 — Sequences for the MSP primer and performing conditions. The table shows the primer sequences of the genes analyzed with MSP, the performing conditions, and the product size. [file bcr3375-S1.DOCX]

**Additional file 1** Sequences for the MSP primer and performing conditions.

| **Gene** | **Sequence (5' → 3')** | **T_A_** | **Cycles** | **Product**  **size** |
| --- | --- | --- | --- | --- |
|  | | | | |
| ***ITIH5* U** | TTGGTGATAGAAATTAAGTAAGTTTGT | 59°C | 40 | 221 bp |
|  | AAAACCACCTATATTAACCCCACA |  |  |  |
| ***ITIH5* M** | TTGGCGATAGAAATTAAGTAAGTTC |  |  | 219 bp |
|  | AACCACCTATATTAACCCCACG |  |  |  |
|  |  |  |  |  |
| ***DKK3* U** | TTTGGTTGGTTAATGGTTGGGTTGTGGTTTTTTTG | 63°C | 38 | 278 bp |
|  | CCCTCACCCACCCCAACTAAACCAAATTACA |  |  |  |
| ***DKK3* M** | GTTGGTTAATGGTCGGGTTGCGGTTTTTTC |  |  | 276 bp |
|  | ACCCACCCCGACTAAACCGAATTACG |  |  |  |
|  |  |  |  |  |
| ***WIF1* U** | GAAGGGTAATTTGTGTTTTTAGTGAGTGTTG | 63°C | 38 | 286 bp |
|  | CCCCCAAAACTACATTCACAATACAATACA |  |  |  |
| ***WIF1* M** | AAGGGTAATTTGCGTTTTTAGTGAGCGTC |  |  | 285 bp |
|  | CCCCGAAACTACATTCACAATACGATACG |  |  |  |
|  |  |  |  |  |
| ***SFRP1* U** | GTTTTGTAGTTTTTGGAGTTAGTGTTGTGT | 60°C | 38 | 125 bp |
|  | CTCAACCTACAATCAAAAACAACACAAACA |  |  |  |
| ***SFRP1* M** | TGTAGTTTTCGGAGTTAGTGTCGCGC |  |  | 136 bp |
|  | CCTACGATCGAAAACGACGCGAACG |  |  |  |
|  |  |  |  |  |
| ***SFRP2* U** | TTTTGGGTTGGAGTTTTTTGGAGTTGTGT | 58°C | 39 | 146 bp |
|  | AACCCACTCTCTTCACTAAATACAACTCA |  |  |  |
| ***SFRP2* M** | GGGTCGGAGTTTTTCGGAGTTGCGC |  |  | 141 bp |
|  | CCGCTCTCTTCGCTAAATACGACTCG |  |  |  |
|  |  |  |  |  |
| ***SFRP5* U** | GTAAGATTTGGTGTTGGGTGGGATGTTT | 60°C | 39 | 141 bp |
|  | AAAACTCCAACCCAAACCTCACCATACA |  |  |  |
| ***SFRP5* M** | AAGATTTGGCGTTGGGCGGGACGTTC |  |  | 136 bp |
|  | ACTCCAACCCGAACCTCGCCGTACG |  |  |  |
|  |  |  |  |  |
| ***RASSF1A* U** | GGTTGTATTTGGTTGGAGTG | 56°C | 38 | 180 bp |
|  | CTACAAACCTTTACACACAACA |  |  |  |
| ***RASSF1A* M** | GTTGGTATTCGTTGGGCGC |  |  | 160 bp |
|  | GCACCACGTATACGTAACG |  |  |  |

U, unmethylated; M, methylated; T_A_, annealing temperature
