# Supplementary material for: Promoter hypermethylation of the tumor-suppressor genes ITIH5, DKK3, and RASSF1A as novel biomarkers for blood-based breast cancer screening
Source: Breast Cancer Res. 2013 Jan 15;15(1):R4. doi: 10.1186/bcr3375 (PMC3672828; doi:10.1186/bcr3375)
Supplement: Additional file 2 — Sequences for the MethyLight primer and performing conditions. The table shows the primer sequences of the genes analyzed with qMSP, the performing conditions, and the product size. [file bcr3375-S2.DOCX]

**Additional file 2** Sequences for the MethyLight primer and performing conditions.

| **Gene** | **Sequence (5' → 3')** | | **T_A_** | **Cycles** | **Product**  **size** | |
| --- | --- | --- | --- | --- | --- | --- |
| ***ITIH5* M** | GGTAAGAAACGGAGTAGGAGGTTT | | 66°C | 50 | 115 bp | |
|  | AACCACCTATATTAACCCCACGAA | |  |  |  |  |
| ***ITIH5* probe** | 6FAM-ACACCCTCCCATCCTACGACTATAACACTT-BHQ-1 | |  |  |  |  |
|  | | | | | |  |
| ***DKK3* M** | GCGTCGTTTTCGTATTTGTATTCG | | 62°C | 40 | 92 bp | |
|  | CGACTAAACCGAATTACGCTACGA | |  |  |  |  |
| ***DKK3* probe** | 6FAM-CGAACTAAATCTACTCGCTCCCGCCGAAA-BHQ-1 | |  |  |  |  |
|  | | | | | |  |
| ***GAPDH* M** | | GAGGATATAGTTTGGTTTTG | 58°C | 40 | 143 bp | |
|  | | CCTACCTAATAATAATCTTTACTTA |  |  |  |  |
| ***GAPDH* probe** | | 6FAM-ACTCCAATCCCTAACCCTACCTTT-BHQ-1 |  |  |  |  |
|  | | | | | |  |
| ***RASSF1A* M** | ATTGAGTTGCGGGAGTTGG | | 59°C | 40 | 65 bp | |
|  | ACACGCTCCAACCGAATACG | |  |  |  |  |
| ***RASSF1A* probe** | 6FAM-CCCTTCCCAACGCGCCCA-BHQ-1 | |  |  |  |  |

M, methylated; T_A_, annealing temperature
