# Supplementary material for: Promoter hypermethylation of the tumor-suppressor genes ITIH5, DKK3, and RASSF1A as novel biomarkers for blood-based breast cancer screening
Source: Breast Cancer Res. 2013 Jan 15;15(1):R4. doi: 10.1186/bcr3375 (PMC3672828; doi:10.1186/bcr3375)
Supplement: Additional file 3 — Correlation of the methylation level in tissue DNA and cfDNA in paired breast cancer samples. This table provides the correlation and P values between DKK3 and ITIH5 promoter methylation in paired breast cancer tissue DNA and serum cfDNA. [file bcr3375-S3.DOCX]

**Additional file 3** Correlation of the methylation level in tissue DNA and cfDNA in paired breast cancer samples.

|  | ***DKK3* tissue DNA** | | | | |
| --- | --- | --- | --- | --- | --- |
|  | **n^a^** | **unmethylated** | **methylated** | **correlation^b^** | **P-value^c^** |
| ***DKK3* serum cfDNA** | 112 |  |  |  |  |
| **unmethylated** |  | 15 | 60 | **0.276** | **0.003** |
| **methylated** |  | 0 | 37 |  |  |
|  | ***ITIH5* tissue DNA** | | | | |
| ***ITIH5* serum cfDNA** | 112 |  |  |  |  |
| **unmethylated** |  | 29 | 56 | **0.255** | **0.007** |
| **methylated** |  | 2 | 25 |  |  |

^a^Only female patients with primary, unilateral, invasive breast cancer were included; ^b^Pearson product-moment correlation coefficient; ^c^Fisher’s exact test at a two-sided significance level of 0.05.
