# Supplementary material for: Promoter hypermethylation of the tumor-suppressor genes ITIH5, DKK3, and RASSF1A as novel biomarkers for blood-based breast cancer screening
Source: Breast Cancer Res. 2013 Jan 15;15(1):R4. doi: 10.1186/bcr3375 (PMC3672828; doi:10.1186/bcr3375)
Supplement: Additional file 4 — Sensitivity and specificity of breast cancer detection in pre- and postmenopausal women in the test and validation sets. This table provides sensitivity and specificity of ITIH5, DKK3, and RASSF1A methylation in pre- and postmenopausal women in the test and validation sets. In addition, area under the curve (AUC) values for all biomarker candidates are shown in these two strata. [file bcr3375-S4.DOC]

**Additional file 4** Sensitivity and specificity of breast cancer detection in pre- and postmenopausal women in the test and validation set.

|  | **Sensitivity in pre- and postmenopausal women** | | | | | | | | | | | |
| --- | --- | --- | --- | --- | --- | --- | --- | --- | --- | --- | --- | --- |
|  | **Test set^a^** | | | | | | | | | | | |
| **Gene** | **Premenopausal** | | | | | | **Postmenopausal** | | | | | |
|  | **Methylation positive** | **%** | **Methylation negative** | **%** | **P-value^d^** | **AUC**  **(95% CI)** | **Methylation positive** | **%** | **Methylation negative** | **%** | **P-value^d^** | **AUC**  **(95% CI)** |
| ***DKK3*** | 8 of 22 | 36 | 23 of 23 | 100 | 0.033 | 0.682  (0.524-0.840 | 29 of 90 | 32 | 78 of 79 | 99 | 0.001 | 0.655  (0.572-0.737) |
| ***ITIH5*** | 7 of 22 | 32 | 23 of 23 | 100 | 0.062 | 0.659  (0.498-0.820) | 20 of 90 | 22 | 73 of 79 | 92 | 0.108 | 0.572  (0.486-0.659) |
| ***DKK3/***  ***ITIH5^c^*** | 10 of 22 | 46 | 23 of 23 | 100 | 0.008 | 0.727  (0.576-0.879) | 36 of 90 | 40 | 71 of 79 | 90 | 0.001 | 0.648  (0.565-0.731) |
|  | **Validation set^b^** | | | | | | | | | | | |
|  | **Premenopausal** | | | | | | **Postmenopausal** | | | | | |
|  | **Methylation positive** | **%** | **Methylation negative** | **%** | **P-value^d^** | **AUC**  **(95% CI)** | **Methylation positive** | **%** | **Methylation negative** | **%** | **P-value^d^** | **AUC**  **(95% CI)** |
| ***DKK3*** | 8 of 21 | 38 | 21 of 21 | 100 | 0.037 | 0.690  (0.526-0.855) | 33 of 117 | 28 | 112 of 114 | 98 | 0.001 | 0.627  (0.555-0.700) |
| ***ITIH5*** | 3 of 21 | 14 | 21 of 21 | 100 | 0.434 | 0.571  (0.395-0.748) | 16 of 117 | 14 | 107 of 114 | 94 | 0.304 | 0.539  (0.465-0.614) |
| ***RASSF1A*** | 9 of 21 | 43 | 16 of 21 | 80 | 0.192 | 0.619  (0.446-0.792) | 54 of 117 | 46 | 81 of 114 | 71 | 0.010 | 0.599  (0.526-0.673) |
| ***DKK3***  ***ITIH5^c^*** | 11 of 21 | 52 | 21 of 21 | 100 | 0.004 | 0.762  (0.611-0.913) | 44 of 117 | 37 | 105 of 114 | 92 | <0.0001 | 0.647  (0.575-0.718 |
| ***RASSF1A***  ***DKK3***  ***ITIH5^c^*** | 15 of 21 | 71 | 17 of 21 | 81 | 0.003 | 0.769  (0.622-0.916) | 77 of 117 | 66 | 76 of 114 | 68 | <0.0001 | 0.679  (0.610-0.748) |

^a^According to McKinlay et al. [1] we applied postmenopausal status at ≥51 years in the patients and the healthy control group; ^b^From 110 patients of the validation set menopausal status was available. Patients and healthy controls without known status was applied postmenopausal at ≥51 years according to McKinlay et al. [1]; ^c^Either gene methylated; ^d^Fisher’s exact test at a two-sided significance level of 0.05; CI, confidence interval; AUC, area under the curve

1. McKinlay SM, Bifano NL, McKinlay JB: **Smoking and age at menopause in women.** *Ann Intern Med* 1985, **103:** 350-356.
